# Editorial: Smart mobile data collection in the context of neuroscience, volume II

**DOI:** 10.3389/fnins.2023.1259632

**Published:** 2023-07-31

**Authors:** Rüdiger Pryss, Winfried Schlee, Manfred Reichert, Thomas Probst, Berthold Langguth, Myra Spiliopoulou

**Affiliations:** ^1^Institute of Clinical Epidemiology and Biometry, University of Würzburg, Würzburg, Germany; ^2^Institute of Medical Data Science, University Hospital of Würzburg, Würzburg, Germany; ^3^Institute for Information and Process Management, Eastern Switzerland University of Applied Sciences, St. Gallen, Switzerland; ^4^Clinic and Policlinic for Psychiatry and Psychotherapy, University of Regensburg, Regensburg, Germany; ^5^Institute of Databases and Information Systems, Ulm University, Ulm, Germany; ^6^Division of Psychotherapy, Department of Psychology, Paris Lodron University Salzburg, Salzburg, Austria; ^7^Knowledge Management Discovery Lab, Otto-von-Guericke- University Magdeburg, Magdeburg, Germany

**Keywords:** mobile data collection, ecological momentary assessment, neuroscience, medicine, psychology, Patient-Reported Outcome Measures, digital phenotyping, sensors

## 1. New findings compared to the first version of this topic

Smartphone technology and, more generally, smart devices have proven to be effective tools for measuring data *in situ* in medicine, psychology, and neuroscience. In this context, many data collection strategies have evolved, which we focused on in the first issue of this Research Topic. As the first topic expired in May 2021 and we have decided to publish a second edition, some new trends in data collection have become apparent. We would like to discuss these below and show where we think the road will lead if smartphones and smart devices are used on a daily and widespread basis in the context of medical and psychological research. As already mentioned, it is hard to imagine research in medicine, psychology and neuroscience without any use of smart devices. One challenge, however, is actually deriving a health benefit from their use. Users quickly lose motivation to use apps over a longer period of time, and measurements with smartphones, despite many advantages, are also associated with many disadvantages that are difficult to remedy (Schleicher et al., 2023). For example, if the ambient volume is to be measured via the microphone and the person using it is sneezing at the time of recording, this is no longer a representative measurement. Another problem is that smartphones from different manufacturers and operating systems cannot always be operated in the same way or that measurements differ (Kraft et al., 2021). On the other hand, possibilities such as digital phenotyping have been discovered to determine psychological parameters of the smartphone user, for example. Unfortunately, there are still too few technical standards and a need for evidence-based recommendations (Agarwal et al., 2016). Finally, a precise distinction must be made between whether the smartphone is used as Ambulatory Assessment or Momentary Ecological Assessment (EMA) only to collect data or also to deliver interventions (Ecological Momentary Interventions, EMI).

Since the first edition of this Research Topic, however, some new trends can be identified. These trends are reflected to some extent in the submissions to this second edition of the theme. One very general trend is that two survey strategies are particularly popular: Patient-Reported Outcomes Measures (PROMs), collected via smartphones, and Digital Phenotyping (DP). With regard to PROMs, it has been learned that self-reports by using EMA cannot only reduce bias compared to traditional self-report methods, but can also be used to evaluate treatment success as a complementary data source to other commonly used outcome assessments to find evidence-based treatments (Basch et al., 2016). Digital phenotyping is also a trend that can be attributed to the increasing use of smartphones, but can almost be described as a new main road (Onnela, 2021). The two aforementioned trends can be seen in the following submitted and accepted papers on this topic as well:

- Daily Contributors of Tinnitus Loudness and Distress: An Ecological Momentary Assessment Study (Simoes et al.).
- A Circadian Hygiene Education Initiative Covering the Pre-pandemic and Pandemic Period Resulted in Earlier Get-Up Times in Italian University Students: An Ecological Study (Montagnese et al.).

However, there are also other trends that are evident in the second issue of the topic. On the one hand, sensor measurements are becoming more and more important as an objective measurement beyond self-reports. Increasingly powerful sensors require new concepts and enable novel considerations. This is nicely demonstrated in the following two papers:

- New Sensor Concepts: Enhancing mHealth data collection applications with sensing capabilities (Karthan et al.).
- New Ideas on Audio: Identifying languages in a novel dataset: ASMR-whispered speech (Song et al.).

On the other hand, measurements over a longer period of time enable not only studies of spontaneous time, but also of circadian rhythms, as this article on the topic shows:

- A Circadian Hygiene Education Initiative Covering the Pre-pandemic and Pandemic Period Resulted in Earlier Get-Up Times in Italian University Students: An Ecological Study (Montagnese et al.).

Finally, in medical examinations, the smartphone is not only used for the person being examined, but increasingly also for reference persons or nursing staff. In one contribution adopted in this topic for children with developmental disabilities, this is done with the involvement of parents. Such settings promise new possibilities, but also require new information architectures for the development of apps and algorithms.

- Smartphone-based behavior analysis for challenging behavior in intellectual and developmental disabilities and autism spectrum disorder—Study protocol for the ProVIA trial (Geissler et al.).

## 2. Where is or should the journey be headed?

Certain trends are emerging in the use of smart devices in medicine, psychology and neuroscience: (1) sensors are increasingly being used more, (2) the smartphone itself is being used to generate novel data (Digital Phenotyping as a new biomarker), and the affected person using or intervening with the device is being expanded to provide further support in multi-person settings.

What we continue to miss, and what needs more attention, is that smartphone-based studies need to be more guided by clinical need and evidence gaps. Also, broader studies need to use the smartphone to learn more about how we can develop it into an everyday technology in a medical and psychological context, which we are still far from doing.

## 3. Summary

What this second version of the topic on “*Smart Mobile Data Collection in the Context of Neuroscience*” has drawn again, smartphone technology is currently developing very dynamically in the field of medicine, psychology and neuroscience. New trends can already be seen in the second edition, which is of course in the nature of things. However, fusing smartphone technology in widespread manner to find and replicate evidence-based medical and psychological interventions has still not been evident in the second edition of this topic, which we feel has received too little attention in general. We will revisit the topic in subsequent years to see if this orientation has strengthened and then where the journey has been headed.

## Author contributions

All authors listed have made a substantial, direct, and intellectual contribution to the work and approved it for publication.
